# Extracurricular multilateral training incorporating cognitive–motor activities and its effects on motor proficiency, cognitive ability, and problematic internet use in adolescents

**DOI:** 10.3389/fpsyg.2026.1951541

**Published:** 2026-09-29

**Authors:** Rizky Nurulfa, Aija Klavina, Csaba Melczer

**Affiliations:** 1 Laboratory of Sports and Nutrition Research, Riga Stradins University, Riga, Latvia; 2 Faculty of Health Science, Doctoral School of Health Science, University of Pecs, Pecs, Hungary; 3 Faculty of Sports Science and Health, Universitas Negeri Jakarta, Jakarta, Indonesia; 4 Department of Health Promotion and Rehabilitation, Lithuanian Sport University, Kaunas, Lithuania; 5 Klaipėda State College, Higher Education Institution, Klaipeda, Lithuania; 6 Institute of Physiotherapy and Sport Science, University of Pecs, Pecs, Hungary; 7 Szentágothai Research Center Physical Activity Research Group, Pecs, Hungary; 8 Faculty of Health Sciences, Complex Sport Performance Diagnostic and Physiotherapy Research Institute, University of Pécs, Pecs, Hungary

**Keywords:** adolescents, after school sports, cognitive function, excessive internet use, motor skills, multilateral training, physical education

## Abstract

**Background:**

After-school physical activity programs may enhance adolescents’ motor and cognitive development, but evidence regarding their effects on problematic internet use (PIU) remains limited.

**Objective:**

This study examined the effects of a 12 week extracurricular multilateral training (EMT) program incorporating cognitive–motor activities on motor proficiency (MP), cognitive ability (CA), and problematic internet use (PIU) among Indonesian adolescents, while accounting for baseline outcomes, daily physical activity, gender, and parental education.

**Methods:**

This parallel-group randomized controlled trial included Indonesian junior high school students participating in a 12-week EMT program delivered twice weekly. MP, CA, and PIU were prespecified as the three primary outcomes and were assessed pre- and post-intervention. ANCOVA compared post-intervention outcomes between the EMT and control groups, adjusting for the corresponding baseline score, daily physical activity, gender, and parental education. Pearson correlations examined associations among baseline outcomes.

**Results:**

The EMT group demonstrated significantly higher post-intervention MP and CA scores and a significantly lower PIU score than the control group after adjustment for the corresponding baseline outcome, daily physical activity duration, gender, and parental education (all *p* < 0.001; ηp^2^ = 0.163–0.422). At baseline, CA was positively associated with MP and negatively associated with PIU in both groups, while MP was negatively associated with PIU only in the EMT group (all *p* < 0.05).

**Conclusion:**

A 12 week extracurricular multilateral training program incorporating cognitive–motor activities improved motor proficiency and cognitive ability and reduced problematic internet use among Indonesian adolescents. These findings support the inclusion of structured, cognitively engaging multilateral training in school-based after-school physical activity programs to promote adolescents’ physical, cognitive, and behavioral health.

## 1 Introduction

Physical activity is a fundamental component of healthy adolescent development, supporting physical growth, neuromuscular coordination, cognitive maturation, and emotional and behavioral regulation. Adolescence is a key developmental stage characterized by rapid physical growth, cognitive maturation, and refinement of emotional and behavioral regulation. During this period, neuromuscular coordination and executive functions develop rapidly, making adolescents highly sensitive to lifestyle influences such as physical activity and digital media use. In recent years, global shifts toward digitalization have been accompanied by reduced physical activity and increased sedentary behaviors, including excessive internet use (World Health Organization, 2020). In Indonesia, these trends are particularly evident, as adolescents increasingly engage in smartphone apps, social media, and online gaming, often replacing movement-based activities (Setyaningsih et al., 2021). This dual burden of low physical activity and high screen exposure highlights the need for school-based interventions addressing both physical and behavioral health outcomes.

Globally, physical inactivity among adolescents remains a major public health issue. Approximately 80% of adolescents fail to meet recommended physical activity guidelines of 60 min of moderate-to-vigorous activity per day (World Health Organization, 2021). This inactivity is associated with increased risks of obesity, cardiovascular disease, metabolic disorders, and poorer mental health outcomes (Guthold et al., 2020). Sedentary lifestyles during adolescence are also linked to reduced long-term physical fitness and health-related quality of life. Similar patterns are observed in Indonesia, where sedentary behavior is increasing among school-aged youth, particularly in urban areas with high digital (Kementrian Kesehatan, 2023). National data show that adolescents frequently exceed several hours of daily screen time, primarily for entertainment and social media use (Rajagukguk et al., 2024). Empirical studies further indicate that higher screen time and lower physical activity are associated with reduced physical fitness and motor performance (Hanifah et al., 2023; Nurulfa and Klavina, 2026). Adolescent development is also influenced by broader family and lifestyle factors, including nutritional status and parental education. Adequate nutrition supports physical growth and cognitive functioning, while parental education may influence health-related behaviors, access to developmental resources, and parental support for physical activity and healthy technology use (Muderedzwa and Matsungo, 2020; Nurulfa et al., 2026b). These findings reflect a growing imbalance between digital engagement and physical activity, with potential implications for motor development.

Motor proficiency is a fundamental component of adolescent development, referring to the ability to perform coordinated and efficient movements. It includes locomotor skills, object control, balance, agility, and reaction ability (Barnett et al., 2012). Higher motor competence is associated with greater participation in physical activity, improved self-confidence, and sustained engagement in active lifestyles (Robinson and Fetterman, 2015). In contrast, lower motor proficiency is linked to reduced physical activity participation and increased sedentary behavior, potentially reinforcing inactivity patterns over time (Lubans et al., 2014). In empirical research, motor proficiency is commonly assessed using standardized instruments such as the Bruininks–Oseretsky Test of Motor Proficiency, Second Edition (BOT-2), which provides a validated evaluation of fine and gross motor skills across multiple domains including coordination, balance, strength, and agility (Bruininks and Bruininks, 2005). This measure is widely used in developmental and physical education research to quantify motor competence in children and adolescents.

Cognitive ability is another essential dimension of adolescent development. It involves attention, memory, decision-making, and executive functioning. During adolescence, maturation of the prefrontal cortex supports improved self-regulation and higher-order cognitive processes (Latino et al., 2021). In this study, cognitive ability was operationalized using Raven’s Standard Progressive Matrices, a nonverbal measure of fluid intelligence that primarily assesses abstract reasoning, pattern recognition, and problem-solving ability with minimal cultural and linguistic bias (Andrade et al., 2001). Physical activity has been shown to enhance cognitive functioning through mechanisms such as increased cerebral blood flow, synaptic plasticity, and neurotrophic stimulation (Hillman et al., 2011; Marques et al., 2018). Evidence suggests that physically active adolescents demonstrate better cognitive outcomes, including improved executive function, attention, and academic performance (Álvarez-Bueno et al., 2016; Donnelly et al., 2016a). Indonesian studies report similar patterns, where physically active adolescents tend to show better cognitive and academic performance than their less active peers (Burhaein, 2017; Yusuf et al., 2021). These associations may be explained by the cognitive demands of complex movement tasks, which require planning, adaptation, and rapid decision-making (Silva et al., 2020). As a result, training programs that integrate physical and cognitive challenges may provide additional developmental benefits.

Alongside these developmental domains, problematic internet use (PIU) has emerged as a growing behavioral concern among adolescents. It refers to excessive, compulsive, or poorly controlled internet use that is difficult to regulate and that leads to significant impairment or distress in daily functioning, including social, academic, and psychological domains (Ioannidis et al., 2019). In adolescent populations, this condition is increasingly relevant because digital platforms are deeply embedded in everyday routines, including communication, entertainment, gaming, and learning activities (Moreno et al., 2022). Importantly, problematic internet use is not only a behavioral pattern but also reflects dysregulated coping and reward-seeking behavior in response to digital stimuli. It is associated with broader developmental consequences, including disrupted sleep patterns, reduced physical activity participation, impaired attention control, emotional difficulties, and lower academic engagement.

Indonesia has experienced rapid growth in internet penetration, exceeding 79%, with adolescents among the most active users (Hanifah et al., 2023). Concerns regarding digital overuse, online addiction, and cyber risks among youth have also increased (Yıldız Durak, 2020). Evidence further suggests that problematic internet use may negatively influence cognitive processes such as attention control, working memory, and impulse regulation (Kim and Lee, 2021). Reduced participation in physical and social activities due to excessive screen time may further contribute to developmental imbalance. In this context, school physical education (Pendidikan Jasmani, Olahraga, dan Kesehatan) represents a key setting to promote healthier activity patterns, although its implementation is often limited by time constraints, teacher capacity, and resource disparities (Mustafa and Dwiyogo, 2020). Multilateral training (MT) may offer a practical, curriculum-aligned approach to strengthen movement experiences within these constraints and support adolescent motor development and balanced activity engagement(Employment and Social Development Canada, 2017; Fischetti and Greco, 2017).

Multilateral training is a comprehensive training approach that promotes the simultaneous development of multiple physical and motor capacities, including coordination, balance, agility, strength, speed, flexibility, and endurance through diverse and progressively challenging movement experiences *(*Bompa and Buzzichelli, 2015). Unlike early sport specialization, multilateral training promotes broad physical literacy and long-term motor development. It is considered particularly appropriate during adolescence, when exposure to varied movement experiences may enhance neuromuscular adaptation and support cognitive engagement through complex motor learning processes (Mahinrad et al., 2020). These principles are reflected in the World Athletics Kids’ Athletics framework, which uses age-appropriate, game-based running, jumping, and throwing activities to develop fundamental movement skills, physical fitness, teamwork, confidence, and enjoyment of physical activity. The program emphasizes learning through play and progressively challenging movement tasks, making it a practical application of multilateral training within school and community settings (Gozzoli et al., 2006; World Athletics, 2021a). By integrating diverse motor tasks that require coordination, decision-making, and adaptive responses, multilateral training may support both motor and cognitive development while increasing participation in structured physical activity (Bompa and Buzzichelli, 2019; Lloyd et al., 2022). Although physical activity interventions have demonstrated benefits for motor and cognitive outcomes (Álvarez-Bueno et al., 2016; Donnelly et al., 2016b), evidence regarding their influence on behavioral outcomes, particularly problematic internet use, remains limited. Furthermore, research examining motor, cognitive, and behavioral outcomes simultaneously is scarce, and existing studies linking physical activity and problematic internet use are predominantly cross-sectional (Lopez-Fernandez, 2018; Nurulfa and Klavina, 2026). The extent to which improvements in motor proficiency are associated with changes in cognitive ability and problematic internet use also remains unclear, particularly in after school-based interventions conducted in low- and middle-income countries such as Indonesia.

Therefore, this study aimed to examine the effects of a 12-week extracurricular multilateral training program on motor proficiency, cognitive ability, and problematic internet use among Indonesian adolescents. A secondary objective was to examine the associations among baseline motor proficiency, cognitive ability, and problematic Internet use. This integrated approach provides evidence from a school-based intervention in a rapidly digitalizing adolescent environment.

## 2 Methods

### 2.1 Research design

This study employed a parallel-group, school-based randomized controlled trial (RCT) with pre–post assessments to examine the effects of a 12-week multilateral training intervention conducted twice weekly in 60 min sessions on motor proficiency, cognitive ability, and problematic internet use among Indonesian adolescents (January–April 2026). Assessments were conducted at baseline (pretest) and immediately after the intervention period (posttest). Participants were allocated to either the experimental group (multilateral training) or the control group (standard school physical education) using a computer-generated block randomization procedure via Research Randomizer, a publicly accessible online tool. The intervention was delivered in a school-based setting under the supervision of trained physical education instructors. Due to the naturalistic school environment, complete control over contextual factors (e.g., attendance, academic workload, and extracurricular commitments) was not feasible; therefore, the study represents a pragmatic, school-based randomized controlled trial conducted under real-world conditions.

### 2.2 Participants

A total of 92 junior high school students aged 12–16 years voluntarily enrolled in the study. Participants were recruited from public schools in Jakarta, Indonesia, through school announcements and information sessions, followed by voluntary registration. After school permission, eligibility screening, and completion of informed consent and parental approval procedures, eligible participants were randomly allocated to the extracurricular multilateral training (EMT) group or control group (CG) using computer-generated block randomization. Inclusion criteria were: (1) enrollment in junior high school, (2) age 12–16 years, (3) medical clearance for participation in moderate-to-vigorous physical activity, and (4) willingness to participate in the full 12-week program. Exclusion criteria included musculoskeletal injuries, neurological disorders, cardiovascular conditions, or any other health condition limiting physical activity participation, and participation in any extracurricular sports program.

The EMT intervention was delivered to 46 participants, comprising 23 Grades 7 and 23 Grade 8 students. During the study, one participant in the CG withdrew before the post-intervention assessment, while three participants in the EMT group were excluded for non-adherence, defined as missing more than three intervention sessions. Thus, 88 participants completed the required baseline and post-intervention assessments and were included in the final analytical sample, comprising 43 participants in the EMT group and 45 in the CG (Figure 1). A per-protocol analysis was performed, with EMT participants required to meet the predefined intervention-adherence criterion. The gender distribution was comparable between groups, with boys comprising 51.2% of the EMT group and 48.9% of the CG (see Table 1).

**Figure 1:**
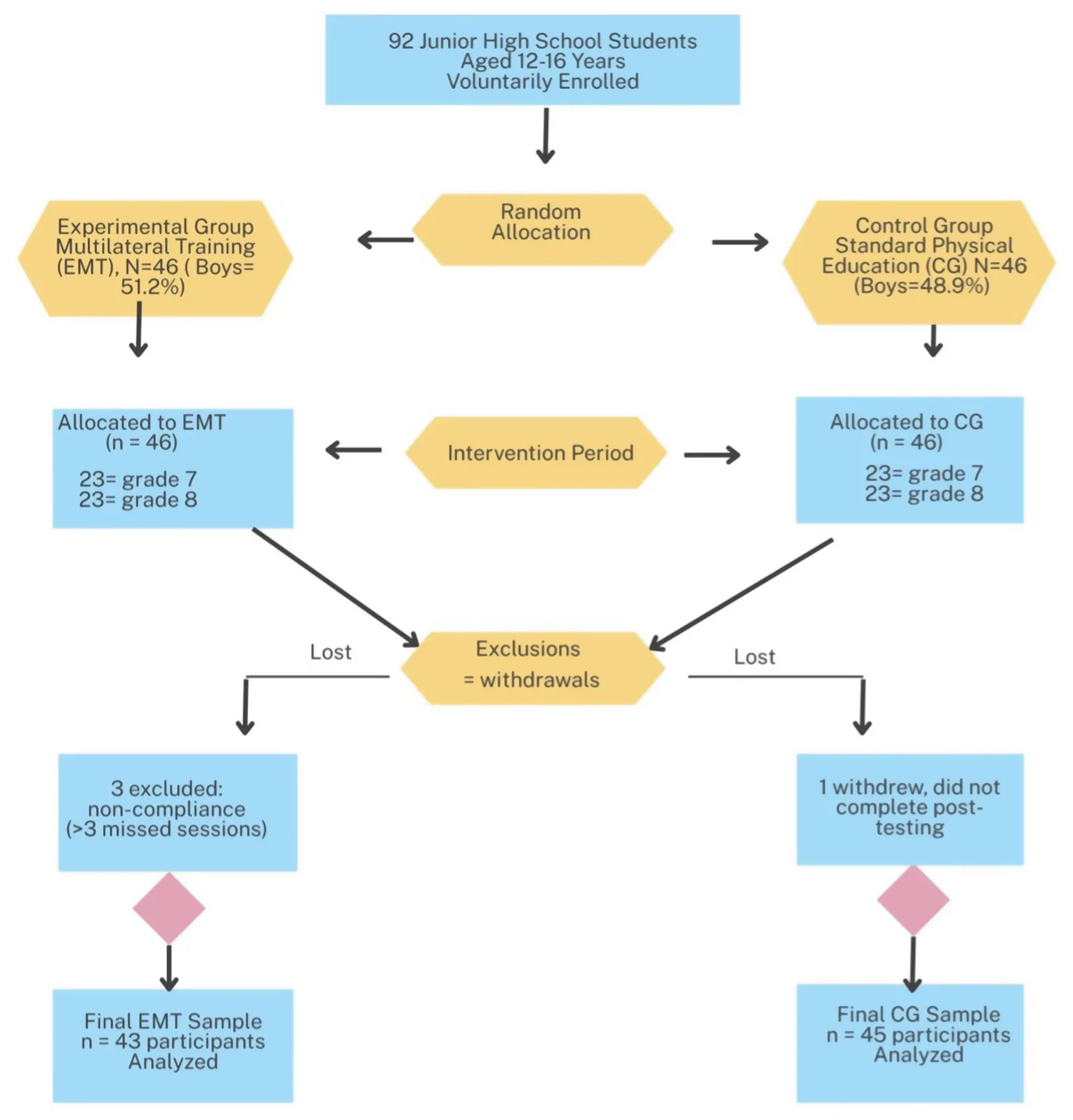
Flowchart of participant enrolment, randomization, follow-up, and per-protocol analysis, resulting in a final sample of 88 adolescents (EMT, *n* = 43; CG, *n* = 45). EMT, extracurricular multilateral training group; CG, control group.

**Table 1:** Baseline demographic and lifestyle characteristics of participant.

Variable	EMT (*n* = 43) mean ± SD, Girls = 48.9%	CG (*n* = 45) mean ± SD, Girls = 51.1%	*p*-value
Age (years)	13.77 ± 1.17	13.78 ± 1.13	0.966
Weight (kg)	50.28 ± 5.32	50.29 ± 4.50	0.993
BMI (kg/m^2^)	20.02 ± 2.19	19.69 ± 2.20	0.477
Height (cm)	158.42 ± 6.36	160.13 ± 5.54	0.180
Weekly physical activity (min/week)	163.95 ± 54.91	188.00 ± 105.78	0.183
Daily physical activity (min/day)	56.51 ± 17.88	59.78 ± 20.20	0.425
Screen time-playing games (h/day)	1.35 ± 0.97	1.67 ± 1.15	0.224
Social media use (h/day)	2.33 ± 1.19	2.31 ± 0.90	0.949
Screen time-study (h/day)	4.86 ± 0.80	4.42 ± 1.34	0.068
Parents’ education level			
Low	6 (14.0)	8 (17.8)	0.695
Medium	19 (44.2)	16 (35.6)	
High	18 (41.9)	21 (46.7)	

Statistical significance was set at *p* < 0.05. EMT, extracurricular multilateral training group; CG, control group; BMI, body mass index; SD, standard deviation.

The study was conducted in accordance with the Declaration of Helsinki, and ethical approval was obtained before data collection. Prior to participation, written informed consent was obtained from the parents or legal guardians of all participants, and all participants provided informed assent. Participation was voluntary, and participants were informed of their right to withdraw from the study at any time without consequence.

### 2.3 Intervention program

#### 2.3.1 Experimental group: multilateral training incorporating cognitive–motor activities

##### 2.3.1.1 Intervention design

Participants allocated to the experimental group completed a 12-week Extracurricular Multilateral Training (EMT) program based on the World Athletics Kids’ Athletics framework and long-term athletic development principles (IAAF, 2016; World Athletics, 2021b). Cognitive engagement was incorporated into each session through cognitive–motor games requiring simultaneous movement execution and information processing. Activities included whistle start–stop games, color and number response tasks, movement imitation, memory sequences, direction-selection tasks, decision-making relays, rule-switch games, inhibition tasks, and Stroop-like movement activities. The difficulty of these tasks was adjusted by modifying stimulus complexity, response time, rules, and dual-task requirements according to participant progress. The intervention consisted of 24 supervised sessions, conducted twice weekly on non-consecutive days after school. Because the experimental group comprised 46 participants, each training day was delivered in two separate sessions of 23 participants to ensure safe supervision and an appropriate coach-to-participant ratio. Each session lasted 60 min, including a 10 min warm-up, 45 min multilateral training component, and 5 min cool-down.

##### 2.3.1.2 Session structure and training delivery

Training was organized into six progressive two-week blocks. During Weeks 1-2, participants performed fundamental running, jumping, throwing, balance, and reaction activities, including sprint relays, standing long jumps, foam javelin throws, single-leg balance, and visual reaction drills. Weeks 3-4 introduced obstacle relays, standing triple jumps, medicine ball chest passes, and cognitive-motor game activities (CMGA) incorporating movement-memory tasks. During Weeks 5-6, participants progressed to multidirectional sprint drills, hurdle relays, ladder jumps, rotational medicine ball throws, and dual-task exercises combining locomotor movements with cognitive challenges. Weeks 7-8 emphasized speed and explosive power through speed ladder relays, repeated jump circuits, overhead medicine ball throws, and decision-making games. In Weeks 9-10, endurance relays, bounding drills, precision throwing, and executive-function tasks were incorporated. The final phase (Weeks 11-12) integrated running, jumping, throwing, agility, balance, and cognitive tasks into competition-style team circuits designed to simulate real-game situations while maximizing movement variability.

Each session began with a standardized warm-up comprising 2 min of light jogging, 3 min of dynamic mobility exercises (leg swings, arm circles, lunges, and high knees), 3 min of coordination drills (agility ladder patterns, cone stepping, lateral shuffles, and skipping), and 2 min of visual- or auditory-based reaction activities. The 45-min training component was delivered in a circuit format consisting of four to six stations, with participants rotating every 5–8 min depending on exercise complexity. Running stations included sprint, shuttle, hurdle, obstacle, Formula One, and endurance relays. Jumping stations incorporated standing long jumps, standing triple jumps, rope jumps, ladder jumps, frog jumps, cross-hopping, and bounding exercises. Throwing stations included foam javelin throws, target throws, medicine ball chest passes, overhead throws, and rotational throws. Additional stations incorporated agility ladder drills, cone-based change-of-direction tasks, single-leg balance exercises, bodyweight strength exercises (e.g., squats, push-ups, planks, and lunges), and obstacle courses. Participants trained in small groups of three to four students per station to maximize active participation while allowing coaches to provide individualized feedback on technique and safety. Exercise difficulty was progressively increased throughout the intervention by manipulating movement complexity, speed, coordination demands, cognitive dual-task requirements, and training volume while maintaining age-appropriate intensity. All sessions were supervised by two World Athletics Level 1-certified coaches (one male and one female) with extensive experience in youth athletics, assisted by members of the research team responsible for session organization, attendance monitoring, and training fidelity.

Exercise intensity was monitored using the Children’s OMNI Rating of Perceived Exertion (0–10 scale). Participants were familiarized with the scale before the intervention and instructed to maintain the prescribed intensity during the main training component. Intensity progressed from RPE 5 during Weeks 1-2, RPE 5-6 during Weeks 3-4, RPE 6 during Weeks 5-6, RPE 6-7 during Weeks 7-8, RPE 7 during Weeks 9-10, and RPE 7-8 during Weeks 11-12. Coaches monitored perceived exertion through regular verbal feedback and adjusted exercise difficulty, repetitions, recovery periods, or task demands when required.

The final 5 min of each session consisted of a low-intensity cool-down (OMNI RPE 2–3) including approximately 2 min of slow walking or light movement followed by 3 min of stretching and recovery activities targeting major muscle groups. To maintain adolescent engagement, different recovery formats were applied throughout the intervention, including individual static stretching, partner-assisted stretching, music-based relaxation stretching, mobility-flow exercises, breathing exercises, and group stretching routines. Individual stretching was emphasized during Weeks 1–4, partner-based recovery activities were introduced during Weeks 5–8, and music-supported stretching, mobility sequences, and guided relaxation activities were incorporated during Weeks 9–12. Research team members assisted with session organization, attendance monitoring, and intensity recording to ensure standardized implementation. Attendance was recorded at each session, and participants were required to complete at least 80% of sessions (≥19 of 24). Details of the training program are provided in Supplementary Table 1.

##### 2.3.1.3 Training progression

Progressive overload was systematically applied throughout the 12-week intervention by progressively increasing motor, physical, and cognitive demands across six two-week training blocks. During Weeks 1-2, participants focused on familiarization with fundamental movement skills, basic coordination, balance, agility, and object-control activities. During Weeks 3-4, task complexity increased through more advanced relay activities, obstacle courses, jumping, and throwing tasks. During Weeks 5-6, training progressed to multidirectional movements, faster execution, and dual-task activities combining motor performance with decision-making. During Weeks 7-8, participants performed higher-intensity activities emphasizing speed, agility, explosive power, and more complex cognitive-motor challenges. During Weeks 9-10, advanced integrated movement tasks were introduced, including faster reactions, greater coordination demands, precision skills, and reduced recovery periods. During Weeks 11-12, participants completed complex athletics-based circuits integrating running, jumping, throwing, agility, balance, and cognitive challenges. Progression was guided by movement quality, task completion, coordination proficiency, and safe execution under coach supervision. This progression model aimed to promote continuous adaptation, motor learning, and cognitive engagement in accordance with long-term athletic development principles (Lloyd et al., 2015; Pesce et al., 2016).

#### 2.3.2 Control group: standard school physical education

Participants in the CG continued their regular physical education according to the standard national school curriculum for the 12-week study period. Sessions were delivered by physical education teachers with more than 10 years of teaching experience and typically consisted of 2 × 40 minute lesson per week, including conventional warm-up exercises, recreational sports, and general physical fitness activities. No structured multilateral training, athletics-specific exercises, or other experimental intervention components were introduced during the study period. Details of the lesson program are provided in Supplementary Table 2.

### 2.4 Instruments and measurement procedures

#### 2.4.1 Motor proficiency assessment

Motor proficiency was assessed using selected subtests from the Bruininks–Oseretsky Test of Motor Proficiency, Second Edition (BOT-2). The BOT-2 is a standardized instrument designed to assess gross and fine motor skills in children and adolescents aged 4–21 years and has demonstrated high reliability and validity across multiple (Venetsanou et al., 2016). The BOT-2 has also been widely used in sports science and pediatric exercise research to evaluate motor competence and physical performance. Five BOT-2 subtests were selected in this study: Bilateral Coordination (7 items), Balance (9 items), Running Speed and Agility (5 items), Strength (5 items), and Upper Limb Coordination (7 items). The total administration time ranged between 35 and 45 min per participant. Raw scores from each BOT-2 subtest were converted into standardized point scores according to age-specific normative tables provided in the BOT-2 manual (Bruininks and Bruininks, 2005). Higher scores indicated better motor proficiency performance (Klavina et al., 2022).

#### 2.4.2 Cognitive ability assessment

Cognitive ability was assessed using the Raven’s Standard Progressive Matrices (SPM) test. The Raven SPM is a nonverbal intelligence assessment designed to measure abstract reasoning, logical thinking, and general cognitive ability while minimizing language and cultural bias (Kramer and Huizenga, 2023). The instrument is widely used for adolescent cognitive assessment in educational and psychological research.

The test consisted of 60 multiple-choice items divided into five sections (A–E), with increasing levels of difficulty. Participants were instructed to identify the missing part of visual matrix patterns from six to eight possible alternatives. Testing was conducted collectively in classroom settings under standardized supervision. Raw scores can be interpreted as follows: 0–20 (below average), 21–30 (low average), 31–40 (average), 41–50 (high average), and 51–60 (superior). The RSPM has been validated across cultures and age groups, including adolescents in Southeast Asia, and is suitable for cross-cultural research because it minimizes language and educational biases (Natanael et al., 2023; Scalisi et al., 2020). Participants completed the assessment within 40–45 min. Each correct answer received one point, resulting in a maximum possible score of 60. Higher scores reflected higher levels of cognitive functioning and reasoning ability.

#### 2.4.3 Problematic internet use assessment

Problematic internet use was measured using the Problematic and Risky Internet Use Screening Scale (PRIUSS). The PRIUSS is a validated self-report instrument developed to identify problematic internet behaviors among adolescents and young adults (Jelenchick et al., 2016). The instrument consisted of 18 items categorized into three domains: (1) Social Impair ment, (2) Emotional Impairment, and (3) Risky/Impulsive Internet Use. Participants rated each statement using a 5-point Likert scale ranging from 0 (Never) to 4 (Very Often). The questionnaire required approximately 10–15 min to complete. Total PRIUSS scores were calculated by summing all item responses, with higher scores indicating greater problematic internet use severity. Previous studies have reported strong internal consistency reliability for the PRIUSS among adolescent populations (Jelenchick et al., 2015; Nurulfa et al., 2026a). The translated versions were subsequently pilot tested to evaluate clarity, comprehensibility, and cultural appropriateness for Indonesian adolescents before data collection.

#### 2.4.4 Demographic and healthy lifestyle behavioral

Demographic and health-related behavioral characteristics were collected using items adapted from the Health Behavior in School-aged Children (HBSC) questionnaire (Currie and Widom, 2010). Participants reported their age, gender, parental education level, physical activity participation, and screen-based behaviors. Anthropometric measurements, including body weight and height, were obtained using standardized procedures and used to calculate body mass index (BMI; kg/m^2^). Physical activity behavior was assessed by recording the frequency of participation in physical activity (times per week) and the average duration of daily physical activity (minutes per day) during the previous week (Maniaci et al., 2023).

Screen-based behaviors were assessed by asking participants to report the average number of hours per day spent playing online games, using social media, and engaging in study-related screen activities. These variables were included to characterize participants’ lifestyle behaviors and digital media use patterns. Parental educational attainment was collected as an indicator of family educational background and socioeconomic context (Farias et al., 2023). Participants reported the highest educational level completed by their mother and father using predefined educational categories. Educational attainment was coded on a 5-point scale (1 = primary education or less, 2 = lower secondary education, 3 = upper secondary education, 4 = bachelor’s degree or equivalent, and 5 = postgraduate degree). Maternal and paternal education scores were averaged to create a parental education score and categorized as low (1.00–2.49), medium (2.50–3.49), or high (3.50–5.00) educational attainment. This variable was used to represent participants’ family educational background and socioeconomic context (Bradley and Corwyn, 2002).

### 2.5 Data collection procedures

Data collection was conducted in two phases: pre-test and post-test. Pretest measurements were administered 1 week before the intervention period, while post-test measurements were conducted within 1 week after completion of the 12 week intervention. To minimize participant fatigue, assessments were conducted over two separate days. On the first day, motor proficiency assessments were administered in the school sports hall. On the second day, participants completed the Raven SPM and PRIUSS questionnaires in classroom settings. All assessments were supervised by trained researchers and physical education professionals. Standardized instructions, equipment, and environmental conditions were maintained throughout testing to ensure consistency and reliability of measurements.

### 2.6 Data analysis

Data were analyzed using IBM SPSS Statistics version 27. Descriptive statistics were calculated for all study variables. Data were screened for missing values, outliers, and normality. Normality was assessed using the Shapiro–Wilk test, and homogeneity of variances was assessed using Levene’s test. The assumptions for the planned parametric analyses were considered acceptable. Baseline group differences were examined using independent-samples *t*-tests. Paired-samples *t*-tests were used to describe within-group pre–post changes and were not used to determine the primary intervention effect. Cohen’s *d* was calculated to describe within-group effect sizes, with values of 0.20, 0.50, and 0.80 interpreted as small, moderate, and large effects, respectively.

The three study outcomes were pre-specified as motor proficiency (MP), cognitive ability (CA), and problematic internet use (PIU). The primary intervention effects were evaluated using separate analysis of covariance (ANCOVA) models comparing post-intervention MP, CA, and PIU between the EMT and control groups. Each model included the corresponding baseline outcome as a covariate and adjusted for daily physical activity duration, gender, and parental education. The primary statistical test was the between-group intervention effect in each ANCOVA model. Adjusted estimated marginal means, adjusted between-group mean differences, and 95% confidence intervals (CIs) were calculated. Partial eta squared (ηp^2^) was used as the effect-size measure, with values of 0.01, 0.06, and 0.14 interpreted as small, medium, and large effects, respectively.

As a secondary exploratory analysis, Pearson product–moment correlation analyses were conducted separately for the EMT and control groups to examine associations among baseline MP, CA, and PIU scores before the intervention. Statistical significance was set at *p* < 0.05 (two-tailed). Missing observations were handled using listwise deletion for each analysis.

### 2.7 Ethical considerations

This study was conducted in accordance with the Declaration of Helsinki of the World Medical Association. Ethical approval from the Latvian Academy of Sports Education (No. S/51813, dated January 20, 2024) was obtained as part of the broader research framework and supported the subsequent development of the research conducted in Indonesia. The present school-based intervention study was reviewed and approved by the Ethics Committee of Jakarta State University, Indonesia (No. 104/UN39.14. PT.01.05/1/2026). Written informed consent was obtained from the parents or legal guardians of all participants, and assent was obtained from the participating adolescents.

## 3 Result

Table 2 presents the baseline and post-intervention comparisons of motor proficiency outcomes in the EMT and CG. Significant within-group improvements were observed across all motor proficiency outcomes in the EMT group (all *p* < 0.001), with moderate-to-large effect sizes (*d* = 0.58–1.43). In the CG, significant improvements were found for bilateral coordination (BC), balance (BL), strength (ST), upper-limb coordination (ULC), and overall motor proficiency (MP) (all *p* ≤ 0.007), whereas running speed and agility (RSA) did not improve significantly (*p* = 0.102). Between-group comparisons showed that the EMT group achieved significantly greater improvements than the CG in BC (*p* = 0.001), RSA (*p* < 0.001), ST (*p* = 0.028), and overall MP (*p* < 0.001). No significant between-group differences were observed for BL (*p* = 0.064) or ULC (*p* = 0.155).

**Table 2:** Comparison of motor proficiency sub items scores within extracurricular multilateral training and control groups at pre and post-intervention.

Outcome	Group	Pre-intervention mean ± SD	Post-intervention mean ± SD	Within *p*-value	Mean difference	95% CI of difference	Between *p*-value	Cohen’s *d*
BC	EMT	23.35 (1.53)	25.00 (1.75)	<0.001	1.65	−2.02 to −1.28	0.001	1.38
	CG	23.11 (1.13)	24.04 (1.11)	<0.001	0.93	−1.15 to −0.72		1.30
BL	EMT	27.12 (4.19)	28.40 (3.29)	<0.001	1.28	−1.96 to −0.60	0.064	0.58
	CG	28.60 (5.42)	29.16 (4.99)	0.007	0.56	−0.95 to −0.16		0.43
RSA	EMT	27.05 (3.15)	29.09 (1.90)	<0.001	2.05	−2.66 to −1.43	<0.001	1.03
	CG	32.60 (1.72)	32.89 (1.65)	0.102	0.29	−0.64 to 0.06		0.25
ST	EMT	26.40 (4.54)	27.95 (3.92)	<0.001	1.56	−2.08 to −1.04	0.028	0.92
	CG	28.38 (2.06)	29.31 (2.23)	<0.001	0.93	−1.13 to −0.74		1.43
ULC	EMT	26.81 (4.05)	28.02 (3.73)	<0.001	1.21	−1.73 to −0.69	0.155	0.71
	CG	26.98 (1.60)	27.76 (1.64)	<0.001	0.78	−1.08 to −0.47		0.76

EMT, extracurricular multilateral training group; CG, control group; BC, bilateral coordination; RSA, running speed and agility; ST, strength; ULC, upper limb coordination; CI, confidence interval; SD, standard deviation. Significant effects are indicated at *p* < 0.05.

Table 3 shows that both groups demonstrated significant pre- to post-intervention changes across all three outcomes. In the EMT group, CA increased by 4.23 points (95% CI 3.11–5.35, *p* < 0.001), MP increased by 6.74 points (95% CI 5.29–8.19, *p* < 0.001), and PIU decreased by 2.37 points (95% CI − 2.78 to −1.96, *p* < 0.001). The CG also showed significant changes, with CA increasing by 0.51 points (95% CI 0.23–0.79, *p* = 0.001), MP increasing by 0.93 points (95% CI 0.44–1.43, *p* < 0.001), and PIU decreasing by 0.89 points (95% CI − 1.41 to −0.37, *p* = 0.001).

**Table 3:** Pre- and post-intervention outcomes and within-group changes.

Outcome	Group	Pre-mean (SD)	Post mean (SD)	Within-group *p*	Mean change	95% CI for change
CA	EMT	34.98 (6.09)	39.21 (8.01)	<0.001	4.23	3.11–5.35
	Control	35.69 (6.32)	36.20 (6.68)	0.001	0.51	0.23–0.79
MP	EMT	133.72 (9.81)	140.47 (10.12)	<0.001	6.74	5.29–8.19
	Control	134.89 (9.76)	135.82 (10.19)	<0.001	0.93	0.44–1.43
PIU	EMT	17.37 (5.79)	15.00 (5.43)	<0.001	−2.37	−2.78−−1.96
	Control	18.36 (7.54)	17.47 (7.67)	0.001	−0.89	−1.41−−0.37

Values are mean (SD). Change = post-intervention − pre-intervention. Within-group *p*-values were obtained using paired-samples *t*-tests. CI, confidence interval; CA, cognitive ability; EMT, extracurricular multilateral training; MP, motor proficiency; PIU, problematic internet use. These changes are descriptive and do not represent intervention effects.

Table 4 presents the baseline correlations between cognitive ability, motor proficiency, and problematic internet use, analyzed separately for the EMT group (*n* = 43) and the CG (*n* = 45). In the EMT group, cognitive ability was positively correlated with motor proficiency (*r* = 0.69, *p* < 0.05) and negatively correlated with problematic internet use (*r* = −0.72, *p* < 0.05). Motor proficiency was also negatively correlated with problematic internet use (*r* = −0.75, *p* < 0.05). In the CG, cognitive ability was positively correlated with motor proficiency (*r* = 0.64, *p* < 0.05) and negatively correlated with problematic internet use (*r* = −0.57, *p* < 0.05). The correlation between motor proficiency and problematic internet use was negative but not statistically significant in the CG (*r* = −0.27, *p* > 0.05).

**Table 4:** Baseline correlations between study outcomes cognitive ability, motor proficiency, and problematic internet use by group.

Variable pair	EMT (*n* = 43)	CG (*n* = 45)
CA-MP	0.69*	0.64*
CA-PIU	−0.72*	−0.57*
MP-PIU	−0.75*	−0.27

Pearson correlation coefficients (*r*) were calculated using pre-intervention scores separately for the extracurricular multilateral training (EMT) and control (CG) groups. All tests were two-tailed. *p* < 0.05. CA, cognitive ability; MP, motor proficiency; PIU, problematic internet use; EMT, extracurricular multilateral training; CG, control group.

Table 5 presents the ANCOVA results for post-intervention CA, MP, and PIU after adjustment for the corresponding baseline outcome, gender, daily physical activity duration, and parental education. Significant between-group effects were observed for all three outcomes. For cognitive ability, the group effect was significant, *F* (1, 82) = 48.037, *p* < 0.001, ηp^2^ = 0.369, with higher adjusted scores in the EMT group (39.755 ± 0.405) than in the control group (35.678 ± 0.395), corresponding to an adjusted mean difference of 4.077 (95% CI, 2.907–5.247; *p* < 0.001). For motor proficiency, the group effect was significant, *F* (1, 82) = 59.898, *p* < 0.001, ηp^2^ = 0.422, with higher adjusted scores in the EMT group (141.127 ± 0.528) than in the CG (135.190 ± 0.515), yielding an adjusted mean difference of 5.937 (95% CI, 4.411–7.463; *p* < 0.001). For problematic internet use, the group effect was also significant, *F* (1, 82) = 16.020, *p* < 0.001, ηp^2^ = 0.163, with lower adjusted scores in the EMT group (15.516 ± 0.249) than in the CG (16.973 ± 0.243), corresponding to an adjusted mean difference of −1.457 (95% CI, −2.182 to −0.733; *p* < 0.001). All models were adjusted for the corresponding baseline outcome, daily physical activity duration, gender, and parental education.

**Table 5:** ANCOVA results for post-intervention cognitive ability, motor proficiency, and problematic internet use.

Outcome	Group effect *F*(1, 82)	*p*	Partial *η*^2^	EMT adjusted mean (SE)	CG adjusted mean (SE)	Adjusted mean difference (EMT − CG)	95% CI	Pairwise *p*
Cognitive ability	48.037	<0.001	0.369	39.755 (0.405)	35.678 (0.395)	4.077	2.907–5.247	<0.001
Motor proficiency	59.898	<0.001	0.422	141.127 (0.528)	135.190 (0.515)	5.937	4.411–7.463	<0.001
Problematic internet use	16.020	<0.001	0.163	15.516 (0.249)	16.973 (0.243)	−1.457	−2.182–−0.733	<0.001

Adjusted estimated marginal means (SE) were obtained from ANCOVA models controlling for the corresponding baseline outcome, daily physical activity duration, gender, and parental education. Adjusted mean differences represent EMT minus CG. Pairwise comparisons were Bonferroni-adjusted.

## 4 Discussion

The present study examined the effects of a 12-week multilateral training program on motor proficiency, cognitive ability, and problematic internet use among Indonesian adolescents. Overall, the findings indicate that participants in the EMT group demonstrated higher post-intervention in motor proficiency and cognitive ability and greater reductions in problematic internet use compared with the CG after adjustment for the corresponding baseline outcomes, daily physical activity, gender, and parental education. These findings support the proposed hypotheses and suggest that a structured multilateral training program incorporating cognitive–motor activities may benefit physical, cognitive, and behavioral outcomes in adolescents.

### 4.1 Motor proficiency outcomes

The ANCOVA showed a significant between-group effect on post-intervention motor proficiency, with higher scores in the EMT group than in the CG after adjustment for baseline motor proficiency, daily physical activity, gender, and parental education. The large effect size indicates a meaningful adjusted group difference and suggests that EMT may contribute to higher post-intervention motor proficiency during adolescence. This finding is consistent with developmental models emphasizing adolescence as an important period for refining movement competence and neuromotor coordination (Duncan et al., 2017; Khodaverdi et al., 2016). EMT emphasizes exposure to diverse movement patterns and physical challenges, which may facilitate broader motor skill acquisition than more specialized forms of exercise (Burton et al., 2023; Robinson et al., 2015). The observed improvements are also consistent with previous intervention studies demonstrating that structured physical activity programs can enhance motor competence through repeated practice, task variability, and progressive (Kumar et al., 2015; Logan et al., 2015). Such adaptations may reflect improved neuromuscular coordination, movement efficiency, and motor control developed through regular participation in varied physical activities. The multilateral and progressively structured nature of EMT may have provided repeated opportunities to practice diverse movement skills while adapting to changing task demands. Importantly, the between-group difference remained significant after adjustment for daily physical activity, suggesting that the observed difference may not be attributable solely to differences in the overall amount of physical activity. Instead, the varied, progressive, and structured characteristics of EMT may have provided additional movement-learning opportunities beyond routine physical activity.

Within the Indonesian context, extracurricular programs may be particularly valuable for supporting motor development by providing additional opportunities for structured physical activity beyond the school curriculum. Consequently, school-based multilateral training may represent a feasible strategy for promoting physical development during adolescence (Wilson and Barnett, 2020).

### 4.2 Cognitive ability outcomes

The ANCOVA showed a significant adjusted between-group effect on cognitive ability, with the EMT group demonstrating higher post-intervention cognitive ability than the CG after controlling for baseline cognitive ability, daily physical activity, gender, and parental education. The large effect size indicates a substantial adjusted group difference. These findings are consistent with previous studies reporting positive associations between physical activity participation and aspects of cognitive functioning in adolescents, particularly executive processes and reasoning (Álvarez-Bueno et al., 2016; Donnelly et al., 2016a).

The cognitive demands embedded within multilateral training such as attention allocation, response selection, and movement adaptation may be relevant in explaining these differences (Kramer and Huizenga, 2023). Unlike conventional physical activity in which cognitive demands may be incidental, the present EMT program deliberately integrated cognitive challenges into movement tasks, requiring participants to process information while executing and adapting motor responses. Such combined cognitive–motor demands may provide repeated opportunities to engage attention, working memory, decision-making, and response control during physical activity.

The persistence of the between-group difference after adjustment for daily physical activity is noteworthy. It suggests that the potential cognitive benefits of EMT may not be explained solely by the amount of physical activity performed. Rather, the cognitive engagement and task complexity incorporated into the intervention may have provided additional stimulation relevant to cognitive functioning. However, this interpretation should remain cautious because the underlying cognitive mechanisms were not directly measured in the present study.

However, it should be noted that cognitive ability in this study was assessed using a measure of fluid intelligence (Raven’s Progressive Matrices), which primarily reflects abstract reasoning rather than broader executive functions (Stankov and Schweizer, 2007). Therefore, the observed higher post-intervention Raven-based performance suggests that structured cognitive–motor physical activity may support aspects of fluid intellectual ability, particularly non-verbal reasoning and problem-solving. These findings should be interpreted specifically within the cognitive domain assessed by the Raven’s Progressive Matrices and should not be generalized to academic achievement or comprehensive executive functioning as evidence of improvements in academic achievement or broader executive functioning (Nurulfa et al., 2026a). Future research should incorporate complementary measures of executive functions and academic performance to determine whether these benefits extend to broader educational outcomes. These findings may also be relevant within the Indonesian educational context, where international assessments have identified challenges in higher-order reasoning and problem-solving performance among Indonesian students (Kementrian Kesehatan, 2023; World Health Organization, 2024).

### 4.3 Problematic internet use

After adjustment for baseline PIU, daily physical activity, gender, and parental education, ANCOVA showed a significant between-group effect on post-intervention PIU, with the EMT group having lower adjusted PIU than the CG. The large effect size indicates a meaningful adjusted group difference and suggests that the potential benefits of EMT may extend beyond physical and cognitive outcomes to adolescents’ digital behavior. This finding is consistent with evidence linking physical activity with lower problematic Internet use and suggests that structured exercise may contribute to healthier patterns of digital behavior (Nurulfa and Klavina, 2026).

One possible explanation is that the structured and cognitively engaging nature of EMT provided an alternative to sedentary, screen-based activities while requiring attention, decision-making, response control, and self-regulation (Lopez-Fernandez and Kuss, 2020). The combination of physical, cognitive, and social engagement may therefore have reduced opportunities for prolonged screen use and supported healthier behavioral regulation.

The significant between-group difference after adjustment for daily physical activity is also noteworthy. This finding suggests that the association between EMT participation and lower PIU may not be attributable simply to greater overall physical activity duration. The structured extracurricular setting, regular participation, social interaction, and cognitively engaging activities may have contributed to the observed difference. Nevertheless, these mechanisms were not directly tested in the present study and should therefore be considered hypotheses for future research.

However, PIU is multifactorial and is also influenced by psychological, family, social, and environmental factors (Mihajlović et al., 2023; Spada and Marino, 2017). Thus, the present findings should not be interpreted as indicating that physical activity alone determines or resolves PIU; rather, EMT may represent one component of a broader approach to promoting healthier digital behaviors. Accordingly, integrating structured physical activity with complementary strategies targeting self-regulation, digital literacy, family involvement, and healthy technology use may provide a more comprehensive approach to PIU prevention and management (Venuleo et al., 2021). Future research should investigate the mechanisms underlying these effects and use longer-term follow-up to determine whether reductions in PIU are sustained.

### 4.4 Relationship between motor proficiency, cognitive ability, and problematic internet use

The secondary aim of this study was to examine the relationships among motor proficiency, cognitive ability, and problematic Internet use. Better motor proficiency was associated with higher cognitive ability, supporting the view that motor and cognitive functions are interconnected through shared processes such as attention, planning, decision-making, and information processing (Higashionna et al., 2022; Zhang et al., 2023).

This finding is consistent with evidence linking motor competence with executive and cognitive functioning during childhood and adolescence and with intervention studies showing that coordinative and cognitively engaging physical activities can benefit both motor and cognitive outcomes (Burton et al., 2023). Similarly, intervention studies indicate that coordinative and cognitively engaging physical activities can simultaneously improve motor and cognitive outcomes (Latino et al., 2021). The present findings extend this evidence to Indonesian adolescents and highlight the potential value of multilateral activities that integrate cognitive demands with movement.

Higher motor proficiency and cognitive ability were also associated with lower problematic internet use, particularly among adolescents participating in EMT. Although these associations should not be interpreted as evidence of causal relationships among the three outcomes, they suggest that physical, cognitive, and behavioral domains may be meaningfully interconnected during adolescence. Previous research has similarly linked physical activity and motor competence with healthier behavioral profiles and lower risk of problematic digital engagement (Nurulfa and Klavina, 2026).

### 4.5 Influence of individual and family factors

The adjusted analyses indicated that individual and family factors contributed differently across outcomes. Parental education was particularly relevant to motor proficiency, whereas gender and daily physical activity did not show significant effects across the primary adjusted models. These findings are consistent with evidence that parental educational and socioeconomic characteristics can influence adolescents’ access to developmental resources, physical activity opportunities, cognitive stimulation, and health-related behaviors (Bradley and Corwyn, 2002; Duncan and Magnuson, 2012; Hackman et al., 2015). Higher parental education may also support adolescent development through greater access to learning resources, cognitive stimulation, health literacy, parental monitoring, and guidance regarding technology use (Chen and Fan, 2024; Domoff et al., 2019; Kalmus et al., 2015).

These findings highlight the importance of considering the family context when implementing school-based interventions, as adolescents may have different levels of support and resources beyond the intervention setting. However, because parental education was included as a covariate rather than a primary explanatory variable, the observed association should not be interpreted as evidence of a causal effect of parental education. Overall, EMT may provide a practical school-based approach to supporting motor proficiency, cognitive ability, and healthier digital behavior within the broader developmental and family environment. Future interventions may therefore benefit from incorporating family engagement alongside structured physical activity and strategies that promote healthy digital behaviors.

### 4.6 Practical implications

The findings have several practical implications for adolescent health and education. First, the results suggest that a structured multilateral training program incorporating cognitive–motor activities can be implemented as an extracurricular school-based intervention to support motor proficiency and cognitive ability. The use of varied movement tasks, progressive challenges, and cognitively engaging games may provide a practical alternative to highly specialized training by addressing several developmental domains within the same program.

Second, the lower adjusted post-intervention PIU observed in the EMT group suggests that structured physical activity may contribute to healthier digital behaviors when adolescents are provided with meaningful and engaging alternatives to sedentary screen-based activities. However, physical activity should not be considered a stand-alone solution to problematic internet use. School-based programs may be strengthened by incorporating digital literacy, self-regulation strategies, parental involvement, and guidance on healthy technology use.

Third, the findings highlight the importance of considering family and contextual factors when implementing adolescent interventions. Parental education was significantly associated with motor proficiency, suggesting that intervention delivery may benefit from supportive home environments and communication with parents or caregivers. In addition, nutritional behaviors should be considered in future school-based health programs because dietary intake and nutritional status may influence physical development, cognitive functioning, and general well-being.

### 4.7 Limitations and future directions

Several limitations should be acknowledged. First, because recruitment was feasibility-based, no prospective sample-size calculation was performed. The final analytical sample comprised 88 participants. Although statistically significant adjusted between-group effects were observed for the three primary outcomes, the observed effect sizes and confidence intervals should be interpreted in consideration of the sample size, particularly regarding the detection and precision of smaller effects. Future studies should conduct an a-priori sample-size calculation based on a pre-specified primary outcome and expected effect size to ensure adequate statistical power.

Second, outcomes were assessed immediately after the 12 week intervention, with no long-term follow-up. Thus, the sustainability of improvements in motor proficiency and cognitive ability and reductions in problematic internet use remains unknown. Third, Raven’s Progressive Matrices primarily assesses non-verbal reasoning and fluid intellectual ability; therefore, the findings should not be interpreted as evidence of improved academic achievement or comprehensive executive functioning. Future studies should include broader cognitive and academic measures.

Fourth, nutritional status and dietary intake were not assessed, and their potential influence on the outcomes could not be controlled. Future research should incorporate validated nutritional measures. Finally, problematic internet use is influenced by psychological, family, social, and environmental factors that were not directly targeted by the intervention. Future studies should examine integrated programs combining physical activity with digital literacy, self-regulation, and parental involvement using larger, more diverse samples and longer follow-up periods.

## 5 Conclusion

The present study indicates that a 12-week extracurricular multilateral training program incorporating cognitive–motor activities was associated with higher post-intervention motor proficiency and cognitive ability and lower problematic Internet use among Indonesian adolescents after adjustment for the corresponding baseline outcomes, daily physical activity, gender, and parental education. These findings suggest that a structured, progressive, and cognitively engaging physical activity program may provide benefits across physical, cognitive, and behavioral domains within a school-based extracurricular setting.

The findings should be interpreted cautiously because cognitive ability was assessed using a measure of fluid intellectual ability rather than broader cognitive or academic outcomes. Further research should examine the long-term sustainability of these outcomes and investigate whether combining structured physical activity with family engagement, nutritional support, and strategies promoting healthy digital behavior can provide broader and more sustained benefits for adolescent development.

## Data Availability

The original contributions presented in the study are included in the article/Supplementary material, further inquiries can be directed to the corresponding author.
