# Supplementary material for: Extracurricular multilateral training incorporating cognitive–motor activities and its effects on motor proficiency, cognitive ability, and problematic internet use in adolescents
Source: Front Psychol. 2026 Sep 29;17:1951541. doi: 10.3389/fpsyg.2026.1951541 (PMC13658619; doi:10.3389/fpsyg.2026.1951541)
Supplement: Supplementary file 1 [file Data_Sheet_1.pdf]

Supplementary Material

Supplementary Table S1. Twelve-Week Extracurricular Multilateral Training Incorporating Cognitive–Motor Activities

Weeks	Warm-up (10 min)	Main part: dual tasks activities (45 min; 4–6 stations, 5–8 min/station)		Cool-down (5 min)	Target Intensity (OMNI RPE)
		Multilateral Training	Cognitive–Motor/Game Activities		
**1-2**	Light jogging, dynamic mobility, ladder drills, reaction games	**Running:** Sprint Relay, Shuttle Relay (10–15 m)<br>**Jumping:** Standing Long Jump, Rope Jump<br>**Throwing:** Foam Javelin Throw, Beanbag Target Throw<br>**Motor skills:** Agility ladder, balance beam, cone stepping, bodyweight squats	Stop-go games, whistle reaction, colour recognition, copy-the-leader	Individual recovery cool-down (slow walking + instructor-led static stretching)	5
**3–4**	Same warm-up with increased movement speed	**Running:** Formula One Relay (basic), Obstacle Relay, Shuttle Run<br>**Jumping:** Frog Jump, Standing Triple Jump (introductory)<br>**Throwing:** Medicine Ball Chest Pass, Foam Javelin Accuracy Throw<br>**Motor skills:** Zig-zag running, push-ups, plank, balance tasks	Memory relay, number-calling games, movement sequence recall	Partner stretching cool-down to introduce cooperation and body awareness	5–6
**5–6**	Dynamic warm-up including multidirectional running	**Running:** Formula One Relay, Hurdle Relay, Shuttle Relay with direction changes	Visual cue decision tasks, dual-task running,	Music-based relaxation cool-	6

		**Jumping:** Ladder Jump, Cross Hopping, Standing Triple Jump<br>**Throwing:** Rotational Medicine Ball Throw, Foam Javelin Throw<br>**Motor skills:** Reactive sprints, multidirectional agility ladder, obstacle circuit	movement + memory	down combined with stretching	
**7–8**	Same warm-up with faster reaction drills	**Running:** Sprint Relay, Speed Ladder Relay, Hurdle Relay<br>**Jumping:** Bounce Run, Multiple Jump Circuit<br>**Throwing:** Target Throw (greater distance), Medicine Ball Overhead Throw<br>**Motor skills:** Squat jumps, reactive obstacle course, lateral movements	Decision-making relay, partner mirror games, rapid color-response games	Mobility flow cool-down emphasizing flexibility and movement quality	6–7
**9–10**	Advanced dynamic warm-up with multidirectional reaction tasks	**Running:** Advanced Formula One Relay, Sprint Relay, Endurance Relay<br>**Jumping:** Bounding Drills, Standing Triple Jump, Ladder Jump<br>**Throwing:** Backward Medicine Ball Throw, Precision Foam Javelin Throw<br>**Motor skills:** push-ups, balance circuits	Rule-switch games, inhibition tasks, Stroop-like movement games	Group stretching circle and breathing exercises to support recovery after higher intensity training	7
**11–12**	High-tempo warm-up emphasizing reaction and coordination	**Running:** Mixed Relay Competition, Obstacle Relay Challenge, Endurance Relay<br>**Jumping:** Combined Jump Relay, Bounding Circuit,	Team strategy games, working-memory relay, executive-function challenges, rapid	Participant-choice recovery cool-down (partner stretching, music stretching, or mindfulness	7–8

		Standing Long Jump Competition<br>**Throwing:** Combined Throw Relay (Foam Javelin + Medicine Ball), Target Accuracy Challenge<br>**Motor skills:** Integrated athletics circuit combining running, jumping, throwing, agility and balance	decision-making competition	breathing) to enhance autonomy and motivation	

frontiers

Supplementary Table S2. Physical Education (PJOK) Block Schedule for Control Group based on Indonesian Curriculum During the 12-Week Period.

Week	PJOK Learning Element	Learning Topic/Sub-material	Opening Activities (Pendahuluan)	Main Activities (Kegiatan Inti)	Closing Activities (Penutup)	Duration
1	Fundamental Movement Skills (Terampil Bergerak)	Basic locomotor, non-locomotor, and manipulative movements	Teacher greeting, attendance check, student motivation, explanation of learning objectives, safety instructions, and general warm-up	Running, jumping, throwing, catching, balance, coordination games, and basic movement exploration	Cool-down, reflection on learning experiences, feedback, summary of key points, and assignment/reminder for next lesson	2 × 40 min
2	Athletics (Atletik)	Basic running skills and relay activities	Attendance, learning objectives, safety briefing, dynamic warm-up, and reaction preparation activities	Sprint technique, acceleration, reaction starts, shuttle relay, baton exchange, and teamwork-based running games	Recovery exercises, stretching, student reflection, teacher feedback, and conclusion	2 × 40 min
3	Physical Fitness Development (Kebugaran Jasmani)	Health-related and skill-related fitness components	Attendance, explanation of fitness concepts, goal setting, pulse checking, and warm-up activities	Fitness circuits including strength, endurance, speed, agility, flexibility, balance, and coordination exercises	Recovery activities, evaluation of perceived effort, reflection, feedback, and closing discussion	2 × 40 min
4	Martial Art	Understanding the facts, concepts, and procedures, as well as practicing specific movement activities	Attendance, learning objectives, safety briefing, dynamic warm-up, and reaction preparation activities	Specific stance movements in pencak silat—individually, in pairs,	Recovery activities, evaluation of perceived effort, reflection, feedback, and closing discussion	2 × 40 min

		in martial arts through pencak silat,		or in groups—both stationary and in motion.		
5	Games and Sports (Permainan dan Olahraga)	Invasion games (football/basketball-based activities)	Attendance, explanation of game objectives and rules, team formation, warm-up	Passing, dribbling, shooting, movement without the ball, small-sided games, teamwork, and tactical practice	Cool-down, discussion of teamwork and strategy, feedback, and conclusion	2 × 40 min
6	Games and Sports (Permainan dan Olahraga)	Net/wall games (volleyball/badminton-based activities)	Attendance, learning objectives, safety explanation, movement preparation	Serving, passing, receiving, positioning, badminton games, rule application	Stretching, reflection on skill development, teacher feedback, and closing	2 × 40 min
7	Gymnastics Activities (Aktivitas Senam)	Body control, balance, and floor gymnastics	Attendance, explanation of gymnastics safety, motivation, flexibility warm-up	Basic gymnastics positions, balance exercises, forward roll, backward roll, flexibility exercises, and movement sequences	Cool-down, peer feedback, reflection, correction of techniques, and summary	2 × 40 min
8	Rhythmic Activities (Gerak Berirama)	Rhythmic movement and aerobic activities	Attendance, introduction to rhythm and movement concepts, light aerobic warm-up	Aerobic movements with music, rhythm combinations, coordination routines, and creative group performances	Relaxation movements, reflection, appreciation of group performance, feedback, and closing	2 × 40 min
9	Traditional and Outdoor Activities	Traditional games and cooperative activities	Attendance, introduction to traditional games, explanation of rules, safety preparation	Indonesian traditional games gobak sodor involving running, cooperation challenges, and	Cool-down, discussion of teamwork and cultural values, reflection, and conclusion	2 × 40 min

				outdoor problem-solving activities		
10	Healthy Lifestyle Education (Pola Hidup Sehat)	Physical activity, exercise safety, and recovery	Attendance, health-related questions, explanation of learning objectives, warm-up	Discussion and practice of warm-up, cool-down, exercise intensity, hydration, injury prevention, and healthy lifestyle behaviors	Reflection, student feedback, summary of healthy lifestyle principles, and closing	2 × 40 min
11	Integrated Movement Skills	Combination of athletics, fitness, and sports skills	Attendance, explanation of integrated activity objectives, safety briefing, dynamic warm-up	Integrated circuits combining running, jumping, throwing, agility, balance, fitness tasks, and modified games	Cool-down, performance feedback, reflection on skill integration, and conclusion	2 × 40 min
12	Assessment and Reflection	PJOK performance evaluation	Attendance, explanation of assessment criteria, preparation activities, warm-up	Practical assessment of movement skills, physical fitness, sport techniques, teamwork, and participation	Cool-down, student reflection, teacher evaluation, feedback, and closing of learning block	2 × 40 min
